# Clockwise rotation of perspective view improves spatial recognition of complex environments in aging

**DOI:** 10.1038/s41598-022-23301-x

**Published:** 2022-11-05

**Authors:** Joaquín Castillo-Escamilla, Isabel Carmona, María del Mar Salvador-Viñas, Miguel Frutos-Lorente, Jorge Luis Ordoñez-Carrasco, José Manuel Cimadevilla

**Affiliations:** 1 Department of Psychology, University of Almeria, Almeria, Spain; 2 Health Research Center, University of Almeria, Almeria, Spain

**Keywords:** Neuroscience, Psychology

## Abstract

Deciphering the human spatial cognition system involves the development of simple tasks to assess how our brain works with shapes and forms. Prior studies in the mental rotation field disclosed a clockwise rotation bias on how basic stimuli are perceived and processed. However, there is a lack of a substantial scientific background for complex stimuli and how factors like sex or aging could influence them. Regarding the latter point, it is well known that our spatial skills tend to decline as we grow older. Hence, the hippocampal system is especially sensitive to aging. These neural changes underlie difficulties for the elderly in landmark orientation or mental rotation tasks. Thus, our study aimed to check whether the effect of clockwise and anticlockwise rotations in the spatial recognition of complex environments could be modulated by aging. To do so, 40 young adults and 40 old adults performed the ASMRT, a virtual spatial memory recognition test. Results showed that young adults outperformed old adults in all difficulty conditions (i.e., encoding one or three boxes positions). In addition, old adults were affected more than young adults by rotation direction, showing better performance in clockwise rotations. In conclusion, our study provides evidence that aging is particularly affected by the direction of rotation. We suggest that clockwise bias could be linked with the cognitive decline associated with aging. Future studies could address this with brain imaging measures.

## Introduction

### How does mental rotation work?

When processing spatial relationships, a single viewpoint of a scene is not enough. Knowing how an environment looks from different locations is crucial in interpreting external inputs of a scene correctly. In this regard, a mental rotation transformation starting from the original scene is demanded, which can be achieved by using two different strategies^1,2^. The first one is object-based, where stimuli are rotated in relation to the environment. The second strategy is perspective transformation, where mental rotations of an individual point of view are performed. These strategies are also described as object-mental rotation and subject-mental rotation, respectively^3^. Mental rotation proficiency is positively related to route learning^4,5^ and allocentric map processing^6^, and it is an important skill for creating a spatial relationship between landmarks and building a cognitive map^7^. Current evidence states that mental rotation is a complex ability dependent on the integrity of closely synchronized brain networks, including inferior temporal, ventral, dorsal, prefrontal regions^8^, and intraparietal regions^9,10^.

Regardless of the strategy, mental rotation comprises three distinct phases that are either considered sequential^11^ or slightly overlapped^12^. First is the encoding of stimuli, second is the proper rotation mechanism, and finally is a decision-making process dependent on the task methodology. The latter component is usually assessed under a recognition paradigm, where participants need to identify if a rotated image is equivalent or not to the original one.

### Effect of angular disparity and direction on the rotational performance

Since the starting studies in this field^13^, different evidence suggests a linear relationship between angular rotation, response time and errors^14,15^. A possible explanation for this process is described by the “viewpoint alignment hypothesis”^16^. According to this hypothesis, a misalignment effect could occur while doing a realignment process when comparing two perspectives. That is, the viewpoint the agents physically preserve is mentally reoriented into alignment with the to-be-adopted viewpoint. As a result, some studies reflect that linearly increasing angular disparities between the original and to-be-adopted perspective translate into more errors^5,17^. Also, better response times and accuracy are reported when the novel views of an environment are presented in alignment with previously encoded ones^18^. These effects are present when simple and complex stimuli are available^15^ and the misalignment effect could be mitigated if cues are offered^16^.

Interestingly, the direction of the rotation could modulate the misalignment effect, as certain studies identified that recognition errors and/or response time increased when perspective rotation followed an anticlockwise direction^17,19^. The effect also have a correlate in electroencephalographic data using event-related potentials^20^. Moreover, as shown by functional magnetic resonance^21^, a clockwise advantage seems to be related to hemispheric lateralization, with the right hemisphere being superior with clockwise rotations and the left hemisphere with anticlockwise shifts.

Familiarity with clockwise rotations in our daily lives (i.e., clocks) could also be responsible for this phenomenon, favoring the existence of a bias against anticlockwise shifts^22^. This bias is described as a “perception–action-laterality” hypothesis, comprised of three steps: clockwise perception of the environment, clockwise formation of a mental map, and then turning in the same direction^23^. These sequential steps would be explained as an interaction of the previously described neurogenetic and cultural factors, resulting in most people presenting a clockwise bias. The effect has been proven with tasks using simple, isolated stimuli, like letters or 2D lines^19,20^.

Moreover, the angular disparity between memorized stimuli and targets imposes different rotation strategies by some studies^24–26^. Specifically, these works describe that a mental object rotation is performed in lower angular disparities, while a subject mental rotation is performed in higher angular disparities. Both are also considered dissociable, although highly correlated processes, and could imply different types of judgment errors. Hence, subject mental rotation based on egocentric transformations would mainly involve laterality errors (i.e., mistaking left and right), and object mental rotation would involve errors related to angular disparities. Accuracy is increased in the presence of salient environmental landmarks or external visual cues.^27,28^. Reliance on these external cues is critical for the allocentric orientation strategy, where stimuli are encoded in relation with the environment and not by the viewpoint of the observer^29^. Brain imaging studies have reported that the right medial temporal lobe supports this process^30,31^. Hippocampal involvement in rotational processes was also described in certain studies, where patients with deterioration in this structure had shown difficulties in object location memory when viewpoints were rotated^32^. Moreover, increasing the number of object locations to remember in this situation —thus, a higher memory load— could further affect performance^33^.

### Sex and aging as influential factors for mental rotation skills

Cognitive abilities decline in healthy aging, including spatial memory^34^ and mental rotation skills^35,36^. Some studies report that older adults are slower and less precise than young adults in spatial tasks^37^, potentially because of volume reduction in the hippocampal system^38^. The hippocampus is involved in building accurate allocentric spatial representations, which are related to rotational skills^6^. To implement optimal solutions to solve such tasks, older people tend to rely more on egocentric strategies which are better preserved in this age group^39–42^.

Another factor modulating mental rotation is sex^43–46^ Studies report that sex differences in mental rotation^43,44^. Certain studies reflected different neural activation patterns and rotational strategies in males and females^45^, while others showed that males of any age generally outperformed females in perspective-taking measures^46^. Furthermore, the clockwise rotation bias is more prevalent in females^47^ and could be associated with hormonal levels^48^.

### Rotation bias in simple vs complex stimuli: are there differences?

When assessing the rotation direction (clockwise vs. anticlockwise), many studies used isolated items like letters^20,49,50^, Navon figures^51^, unfamiliar characters^52^ or 2D lines^19^. Only a few studies used complex stimuli and conditions equivalent to the real world for an angular disparity effect^53^ or a rotation bias^54^, but more research is needed to confirm if this is a tendency. Therefore, there is an inconsistency in the findings between simple and complex stimuli, which can be explained in light of the dissociation between the far and reaching space^55^. As both of them involving different strategies^56^ and neural activations^57^, there could be some disparities in rotational performance in the processing of simple stimuli vs. complex environments. Considering this evidence, designing a task capable of successfully measuring rotational abilities in the far space—involving more complex and richer environments—is needed.

Spatial memory research has significantly progressed thanks to technological advancements. New computerized tasks showed a high sensitivity in finding spatial impairments where traditional tasks failed^58,59^. They also use more ecologically valid methods and environments^60^. Regarding this, spatial recognition tasks can be comparable to active or passive navigation in finding spatial orientation differences^61^. They are also less technically demanding and methodically comparable to traditional measures of mental rotation. Following this rationale, the Almeria Spatial Memory Recognition Test (ASMRT) was developed by our research group and applied to different samples^62,63^. Participants are required to identify if one or more boxes changed their position in a virtual room. There are two modalities of presentation: presented in a similar viewpoint to the original image, or with variable degrees of viewpoint shifting, precisely measuring the rotation angles and direction. The presence of landmarks within the room, plus changing perspectives, also favors the allocentric strategy over egocentric solutions. ASMRT has proved its sensitivity in identifying differences due to sex or aging^62,63^.

### Study aims and hypotheses

The main goal of this study is addressing how the rotation direction (clockwise vs. anticlockwise) between a memorized scene and subsequent recognition picture could be affected by aging and sex in a complex environment. Following previous studies^20^, the “viewpoint alignment hypothesis”^16^, and the “perception–action-laterality” hypothesis^23^, we predicted performance to be better under clockwise rotations compared to anticlockwise trials. Anticlockwise rotation between encoded and recognition stimuli should be associated with slower response times or less accuracy^19,20^, especially in the elderly, due to their decreased spatial skills^64^. Also, following the apparent higher clockwise bias in females^47^, we could also expect better performance in males over females in anticlockwise conditions.

## Method

### Participants

Eighty participants (n = 80) took part in the study. Forty participants (n = 40) were psychology students from the University of Almeria (20 males; age range 18–30; M = 20; SD = 2.8), and another forty participants were recruited from the Elders Classes from the same institution (20 males; age range 60–79; M = 69; SD = 5.3). All the participants had a normal of corrected-to normal vision (i.e., using glasses or contact lenses) at the moment of assessment, and their gender identity matched their sexual biology. Exclusion criteria were defined as having less than 26 points in the Mini-Mental State Examination, a formal diagnosis of psychological or psychiatric disorders, consumption of harmful substances like drugs and/or alcohol, head trauma, or any other condition that could affect the results. Information about the procedure and general goal of the study was given to the participants, and they were free to quit the study at any time. The study was approved by University of Almeria Ethical Committee (UALBIO2019/022) and was designed following the requirements of the European Communities Council Directive 2001/20/EC and the Helsinki Declaration for biomedical research with humans.

A post-hoc power analysis with the G*Power software v3.1.9.2^65^ was conducted in order to determine the statistical power of both main and interaction effects (within-between subject factors) showed in our study. With an alpha equal to.05, a medium effect size (d = 0.35), and a total sample size = 80, the analysis revealed a statistical power greater than 0.99. The statistical power of correlations results was greater than 0.94.

### Materials

The spatial memory recognition performance was assessed using the Almeria Spatial Memory Recognition Test (ASMRT)^62^. The presentation of the experimental setting occurred through a virtual environment, simulating a museum room with four walls (with a series of spatial cues displayed on them, i.e., portraits) and nine brown boxes aligned symmetrically in a 3 × 3 array. Some of these boxes were colored in green, representing the target stimuli to work with in all task phases. ASMRT was composed of two difficulty levels, defined by the number of boxes to memorize (one box vs. three boxes), with four trials each. The full test consisted of 8 memory images. Therefore, there were 80 recognition images, half for each difficulty level.

**Figure 1:**
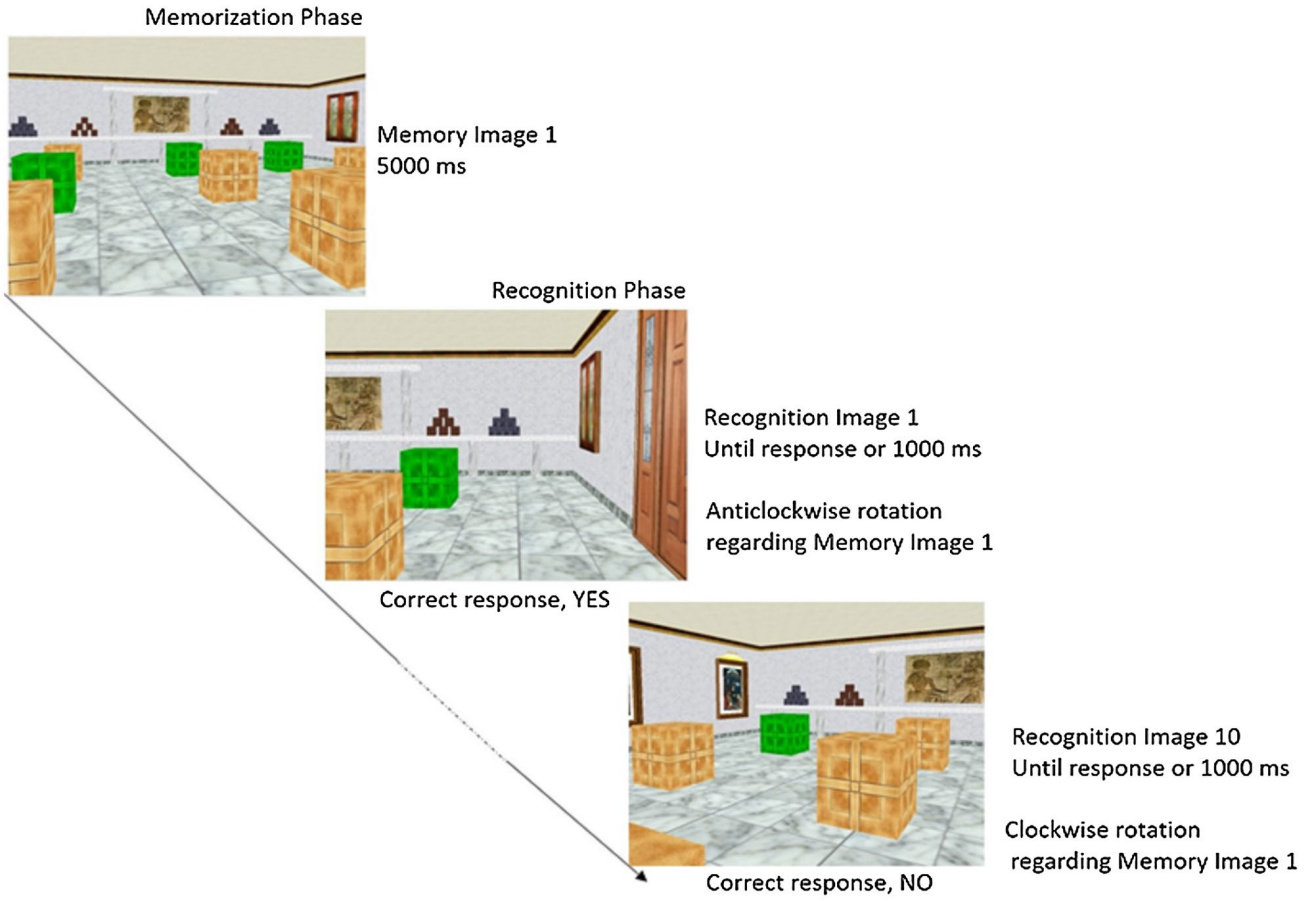
Stimuli sequence (from left to right) of one trials of higher difficulty level (three boxes) of the ASMRT. In the memorization phase, participants need to retain the position of the boxes colored in green. In the recognition phase, ten images were displayed one after another. For each of them, participants needed to identify if the green box was in the same place as the previously memorized. Every recognition image, as seen in the sample images, was in a different orientation when compared with the memory image.

The stimuli sequence of each trial was composed of two distinct phases as follows (see Fig. 1):

- In the “Memorization Phase” participants were shown a picture (memory image) of a virtual room where a total of nine boxes (in a 3 × 3 disposition) were placed. The memory images were displayed for five seconds. All memory images were taken from a first-person viewpoint. Depending on the difficulty level, one or three boxes were colored in green. Participants were asked to remember the position of those green boxes. Three out of four walls of the room contained several stimuli (including a door, a window, or pictures) that could help to disambiguate the spatial locations. Each memory image was taken from a different viewpoint compared to the other.
- In the “Recognition Phase”, a total of 10 images of the same room were presented to the participants one after another. Direction of rotation was manipulated in this phase. Thus, the recognition images were taken from different viewpoints regarding the original image. Only one green box for all difficulty levels was shown in this phase. Participants needed to answer (yes/no) if the green box was in the same position as one of the boxes in the memorized picture. Thus, they had to decide about each image basing on the mental representation they formed during the memory phase under a different viewpoint.

The task was administered in a non-immersive way using a portable computer running the Windows 10 Operating System. The computer was equipped with an Intel Core i5 processor, 4 GB of RAM, and a 15.4 inches LCD screen with a 1920 × 1200 resolution. Through the evaluation, it was connected to a power source in the wall to avoid battery depletion. Additionally, as an internet connection was not demanded during the procedure and/or data collecting, this was disabled through the evaluation.

### Procedure

All participants were tested individually in the University of Almeria institution, and underwent a brief interview to verify the exclusion criteria. They also signed an informed consent before starting the evaluation. After this, ASMRT instructions were presented to them on-screen, followed by a trial example. Then, the experimenter administered the two difficulty levels (memorize the position of one box or three boxes) sequentially in the same order for all participants (one memory image—ten recognition images, four times per difficulty level, with different stimuli in each trial). The participants answered when each recognition image was presented on screen, and the experimenter manually registered their response for each trial to avoid pressure due to a response time limit that could alter performance. The correct responses and errors were collected and treated for each participant and experimental condition in the subsequent data analysis. Participants underwent the full experimental procedure —excluding the initial interview— in a quiet and isolated environment to avoid any external noise or unpredictable intervention that could alter the performance.

#### Angle calculation and distribution in octants

**Figure 2:**
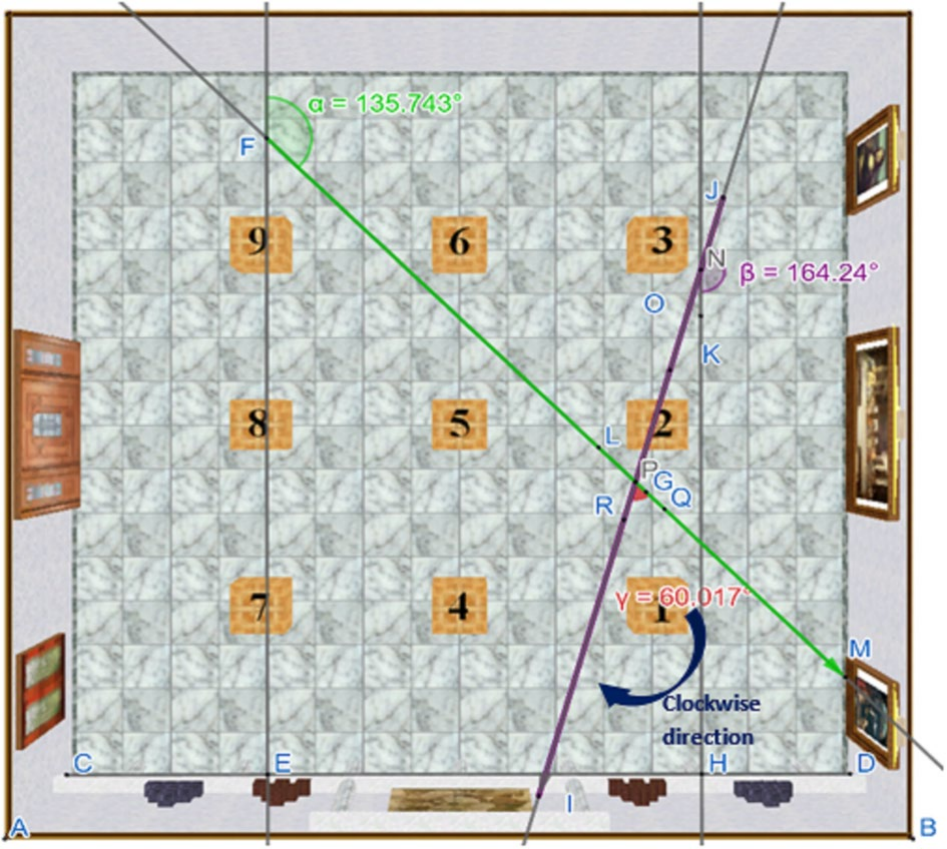
Final step of the angle and direction calculation for the ASMRT. Crossing the angles of the memory image and the recognition image in the zenith plane, we can obtain the difference between both angles (named α—green line—and β—purple line—, respectively) and the direction of the recognition image rotation, representing the exact viewpoint rotation between both images (angle and direction, represented in angle Y).

Euclid’s postulates were employed to calculate the angular difference between the viewpoint of memory images and the correspondent viewpoint of recognition images and their rotation direction (clockwise or anticlockwise). This process assessed whether the angles were homogenously distributed according to the rotation direction in each condition (see Fig. 2). The open-source program GeoGebra Geometry, developed by the Florida Atlantic University, was used for this procedure. ASMRT images were presented for calculations in a conic, two-dimensional, first-person perspective. In this perspective, which is the same experienced by participants, lines are oblique to each other, compared to the zenith perspective, where they would be parallel. The oblique lines going in the same direction also converge in a common vanishing point. The rotation angle is calculated in a conic perspective for each image, and subsequently an angle comparison between memory and recognition images in the zenith perspective is performed in order to ensure maximum consistency.

Firstly, using images in conic perspective, segments based on the floor lines were traced. This method outlined two sets of lines: oblique lines that would be parallel and horizontal in the zenith perspective, and oblique lines that would be parallel and vertical in that same zenith perspective. This was done in order to calculate two independent vanishing points, referenced where each set of lines respectively converged, and used in subsequent calculations. The next step was identifying the medial point of the image width, where a perpendicular line is traced at the midpoint of the image base, obtaining a position vector. There, two segments, originated from the vanishing points calculated before and were connected to the position vector. The junction point was calibrated at a 90º angle, which equals the angle formed by the floor lines in the zenith plane.

When the position vector is crossed with the 90° angle, we can determine the comparison angle between memory and recognition images. It can be the angle formed by the segment of any vanishing points, using floor lines as a guide. In order to maintain consistency in the measurement, the vertical floor segments were used for all images. The angle used for the comparison is the one formed between the position vector and the previously mentioned segment. Depending on the perspective adopted by the correspondent memory image, and to maintain the consistency stated before, the angle to use differs, as GeoGebra Geometry presents the obtuse angle by default. This procedure was initially done for the memory image and subsequently for its corresponding ten recognition images. Figure 2 represents the final result of this process.

**Figure 3:**
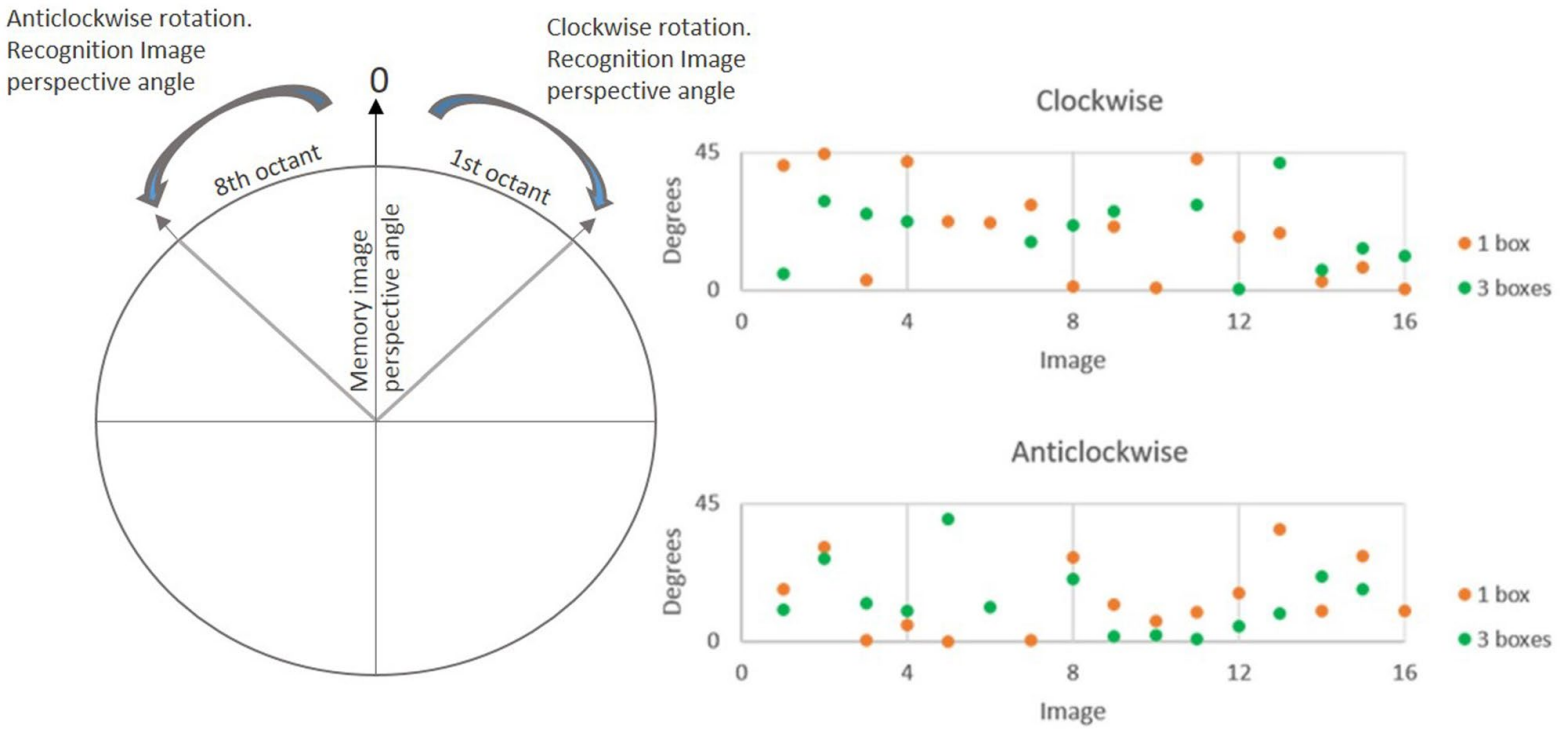
Rotation angle (in degrees) of each viewpoint of recognition image as a function of Difficulty (one box and three boxes) and Direction (clockwise and anticlockwise).

Once the angles and the direction of rotation of each viewpoint of recognition image were obtained, the distribution of the angles was evaluated, observing that most of them were homogeneously distributed between the first and eighth octants, clockwise (first octant: one box, 16 images: M = 34°; SD = 15°; three boxes, 16 images: M = 29°; SD = 12°) and anticlockwise direction (eighth octant: one box, 16 images: M = 32°; SD = 14; three boxes, 16 images: M = 28°; SD = 11°), respectively (see Fig. 3). Only two recognition images in each condition (direction-difficulty) were located unevenly outside these octants, so they were excluded from the analysis. Hence, the total number of trials analyzed was 62 (32 clockwise trials and 32 anticlockwise trials). This allowed controlling of the angular disparity effect on performance.

Moreover, in half of the 16 recognition images for each direction (clockwise and anticlockwise) and for each difficulty level (one box and three boxes), the position of the boxes coincided with the memory image (correct response, yes) and in the other half, they did not (correct response, no). Correct acceptances and correct rejections were registered.

### Statistical analysis

Percentage of correct acceptances (Hits), percentage of incorrect acceptances (100%−%correct rejections, false alarms, FA), and discriminability score (d’ = Z hits rate-Z false alarm rate)^66,67^ were obtained for all participants and experimental conditions. They were analysed in a 2 × 2 × 2 × 2 mixed ANOVA with Age (old and young) and Sex (female and male) as the between-subjects factors, and Difficulty (one box and three boxes), and Direction (clockwise and anticlockwise) as the within-subjects’ factors. Kolmogorov–Smirnov tests were conducted to check the normality of data, and Levene’s tests were used to verify homogeneity of variance. Bonferroni correction was applied to correct for type I error accumulation in multiple comparisons. T-test analyses were conducted to evaluate whether the means were different between conditions, as necessary. Supplementary T-test analyses showed that the angle factor (octant distribution) did not reach significance because all of them were in the same angular region (octant), so it was excluded for the ANOVAs. Analyses were performed with IBM SPSS Statistics v.25 with a significance level of p < 0.050.

## Results

### Hits

The ANOVA (Sex × Age × Difficulty × Direction) showed a main effect of Age (*F*(1, 76) = 51.4; *p* < 0.001; *η*_*p*_^*2*^ = 0.41), Difficulty (*F*(1,76) = 49.8; *p* < 0.001; *η*_*p*_^*2*^ = 0.40), and Direction (*F*(1,76) = 9.3; *p* = 0.003; *η*_*p*_^*2*^ = 0.11]). Young adults were more accurate in their responses than old adults, as can be seen in Table 1. Regarding Difficulty, participants also performed worse when they had to memorize three boxes than one box, and when the stimuli were presented in an anticlockwise direction than when presented clockwise. No other effects were found (*p* > 0.05).

**Table 1:** Mean percentage of Hits for each factor, Age (old and young), Sex (female, male), Difficulty (one box and three boxes) and Direction (clockwise and anticlockwise).

Factor		% Hits: M(SD)	p-value
Age	Young adults	91 (2)	< 0.001
	Old adults	70 (2)	< 0.001
Sex	Female	79 (2)	> 0.050
	Male	82 (2)	> 0.050
Difficulty	One box	85 (1)	< 0.001
	Three boxes	76 (2)	< 0.001
Direction	Clockwise	83 (1)	< 0.010
	Anticlockwise	78 (2)	< 0.010

SD in brackets. (Triple asterisk means p-value < 0.001; double asterisk means p-value < 0.01; ns means p-value > 0.05).

The Difficulty × Age interaction reached significance (*F*(1, 76) = 33.4; *p* < 0.001; *η*_*p*_^*2*^ = 0.31). Further analyses of the interaction revealed significant differences due to Difficulty only for old adults (*F*(1, 38) = 46.8; *p* < 0.001; *η*_*p*_^*2*^ = 0.55). In this group, the Hits percentage was higher in the one box trials (M = 78%, SD = 2) than in the three boxes trials (M = 61%; SD = 2). By contrast, the Difficulty level did not affect performance in young adults (*p* > 0.05), as it was similar between the one box trials (M = 90%, SD = 2) and the three boxes trials (M = 92%; SD = 2).

The Direction x Age interaction was also statistically significant (*F*(1, 76) = 13.9; *p* = 0.005; *η*_*p*_^*2*^ = 0.35). Further analyses of the interaction showed significant differences only in the old adults group (*F*(1, 41) = 8.8; *p* = 0.005; *η*_*p*_^*2*^ = 0.19). For these participants, percentage of Hits was higher in clockwise trials (M = 73%, SD = 3) than in anticlockwise trials (M = 66%; SD = 3). However, performance of young adults was unaffected by direction of rotation (*p* > 0.05; clockwise: M = 92%, SD = 1; anticlockwise: M = 90%; SD = 2).

No other interactions reached significance (*p* > 0.05).

### False alarms

**Figure 4:**
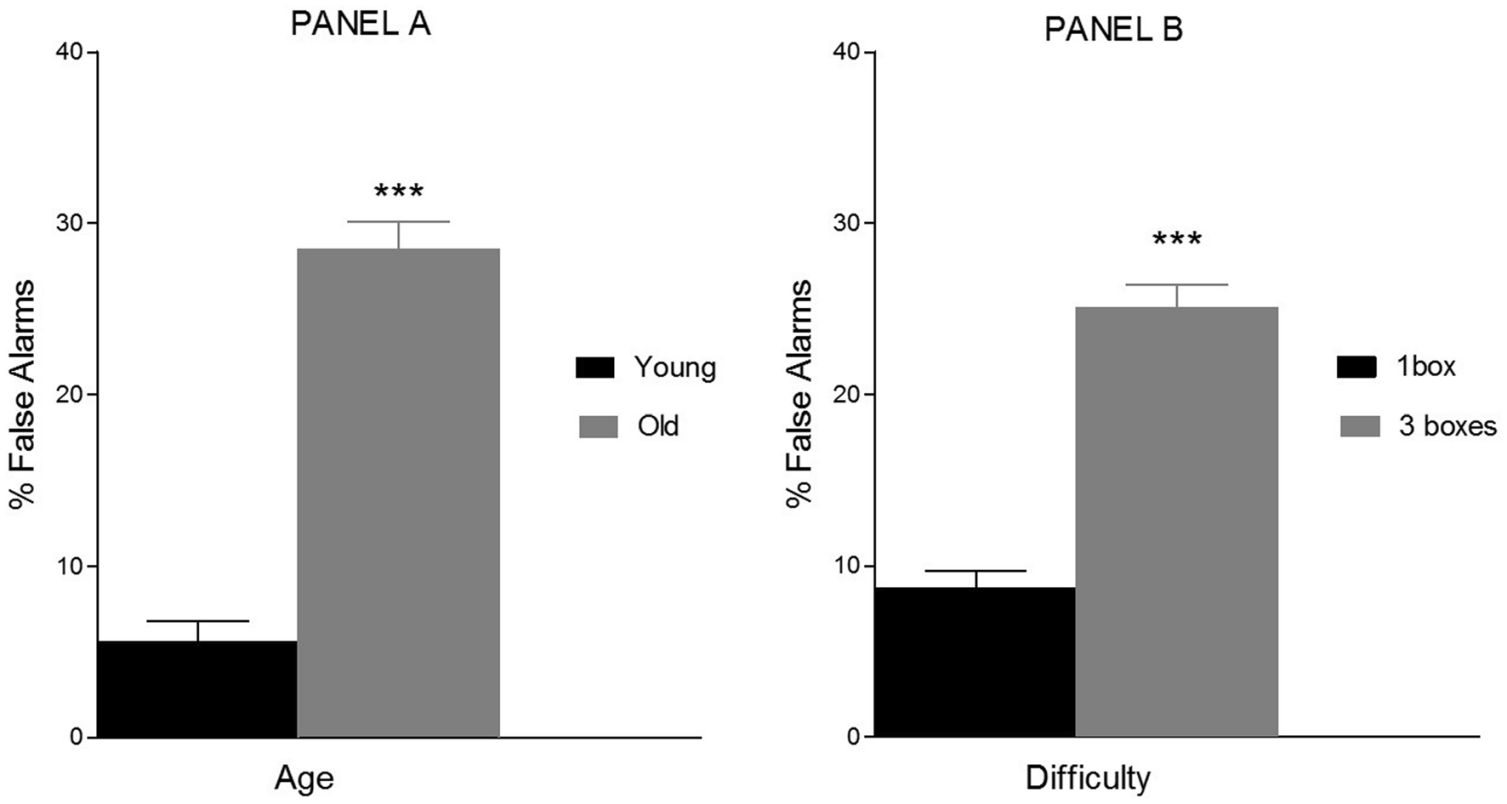
Mean percentage of false alarms as a function of Age (young < 30 years, older > 60 years, Panel **A**) and Difficulty (one box and three boxes, Panel **B**). Triple asterisk means *p*-value < .001.

The ANOVA showed a main effect of Age (*F*(1, 76) = 114.8; *p* < 0.001; *η*_*p*_^*2*^ = 0.60), Sex (*F*(1,76) = 4.4; *p* = 0.039; *η*_*p*_^*2*^ = 0.06) and Difficulty (*F*(1, 76) = 90.1; *p* < 0.001; *η*_*p*_^*2*^ = 0.54). As can be seen in Fig. 4, the percentage of false alarms was higher in the old adults’ group (M = 28%; SD = 2) than in the young adults’ group (M = 5%; SD = 2).

Regarding the Difficulty level, percentage of false alarms was lower in one box trials (M = 8%; SD = 1) than in 3 box trials (M = 25%; SD = 3). Overall, percentage of false alarms was lower in males (M = 14%; SD = 1) than in females (M = 19%; SD = 2). No other main effects were found (*p* > 0.05).

There were significant interactions Difficulty x Age (*F*(1, 76) = 80; *p* < 0.001; *η*_*p*_^*2*^ = 0.57), and Difficulty × Direction (*F*(1, 76) = 11.4; *p* = 0.001; *η*_*p*_^*2*^ = 0.14).

The analyses of Difficulty x Age interaction revealed a significant effect of Difficulty only in old adults (*F*(1, 42) = 96.4; *p* < 0.001; *η*_*p*_^*2*^ = 0.70). The percentage of false alarms was higher in the three boxes trials (M = 44%; SD = 1) than in one box trials M = 12%, SD = 2) in this group.

**Figure 5:**
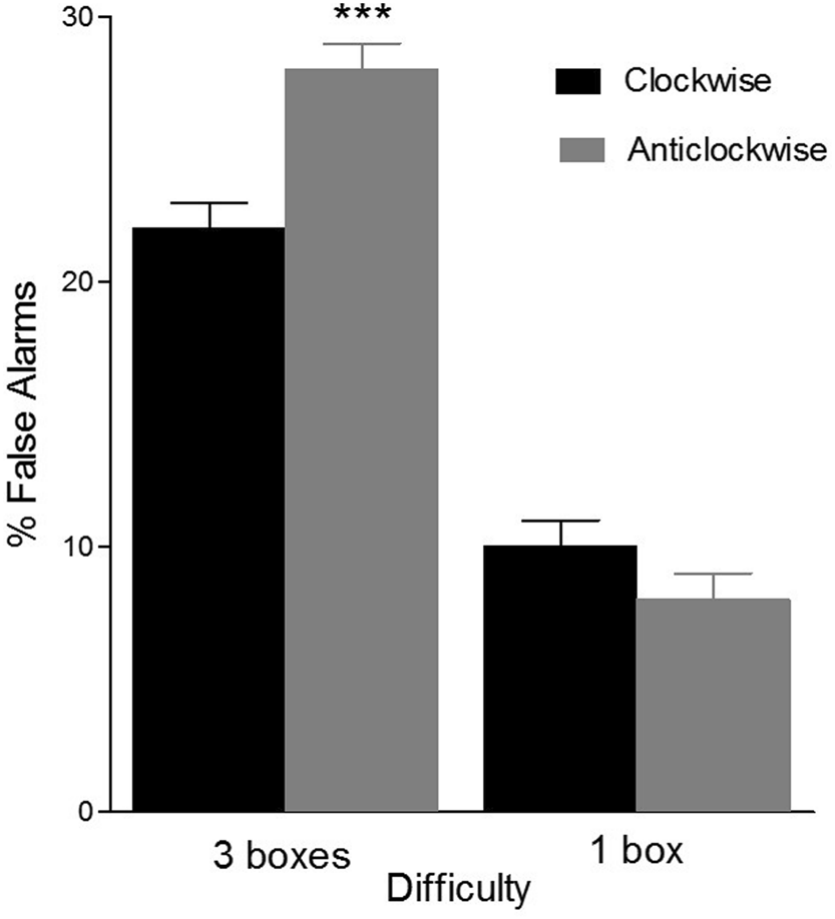
Mean percentage of false alarms as a function of Difficulty (one box and three boxes) and Direction (clockwise and anticlockwise). Triple asterisk means *p*-value < .001.

Finally, the Difficulty × Direction interaction showed significant differences due to Direction in the higher difficulty level (three boxes: *F*(1, 76) = 8.5; *p* = 0.005; *η*_*p*_^*2*^ = 0.11). The percentage of false alarms was higher in anticlockwise trials (M = 27%; SD = 1) than in clockwise trials (M = 22%; SD = 2) (see Fig. 5). By contrast, in one box trials, there was no difference in percentage of false alarms between both directions (*p* > 0.05).

Complementary t-test analyses (see Table 2) revealed that sex modulated the percentage of false alarms in three boxes-anticlockwise trials, only for the young adults’ group (t (38) = 2.2, *p* = 0.038). Differences in percentage of false alarms due to sex for old adults were absent (*p* > 0.05).

**Table 2:** Mean percentage of Hits, False Alarms (FA), and d’ score for each factor, Age (old and young), Sex (female, male), Difficulty (one box and three boxes) and Direction (clockwise and anticlockwise).

Age	Difficulty	Direction	Mean (Female)			Mean (Male)			*p-value* from T-test (Female vs. Male)		
			% HITS	% FA	d' index	% HIT S	% FA	d' index	HITS	FA	d' index
Young adults	One box	Clockwise	87	08	0.79	95	03	0.92	n.s.	n.s.	n.s.
		Anticlockwise	83	05	0.79	95	03	0.93	n.s.	n.s.	n.s.
	Three boxes	Clockwise	91	05	0.78	97	01	0.93	n.s.	n.s.	n.s.
		Anticlockwise	87	12	0.75	94	04	0.89	n.s.	< 0.050	< 0.050
Old adults	One box	Clockwise	83	12	0.51	83	14	0.45	n.s.	n.s.	n.s.
		Anticlockwise	77	10	0.46	68	10	0.52	n.s.	n.s.	n.s.
	Three boxes	Clockwise	63	44	0.38	62	38	0.46	n.s.	n.s.	n.s.
		Anticlockwise	56	52	0.25	62	40	0.24	n.s.	n.s.	n.s.

### Discriminability index

T-test analyses confirmed that the performance in both groups of age was above the chance level in all conditions (see Table 3).

**Table 3:** T-test was conducted to evaluate differences between the discriminability (d’) index and the chance level, as a function of Age (young, < 30 years, and older, > 60 years), Difficulty (one box and three boxes), and Direction (clockwise and anticlockwise). df, degree of freedom.

Age	Difficulty	Direction	T-test (d'score vs. Chance level)		
			*T*	df	*p*-value
< 30	One box	Clockwise	33.4	39	< 0.001
		Anticlockwise	34.2	39	< 0.001
	Three boxes	Clockwise	46.3	39	< 0.001
		Anticlockwise	25.5	39	< 0.001
> 60	One box	Clockwise	11.7	39	< 0.001
		Anticlockwise	13.7	39	< 0.001
	Three boxes	Clockwise	10.9	39	< 0.001
		Anticlockwise	5.1	39	< 0.001

The ANOVA showed a main effect of Age (*F*(1, 76) = 109.6; *p* < 0.001; *η*_*p*_^*2*^ = 0.59), the discriminability index was higher in young adults (M = 0.86; SD = 0.01) than in old adults (M = 0.42: SD = 0.03); Sex (*F*(1,76) = 3.8; *p* = 0.049; *η*_*p*_^*2*^ = 0.10), where, overall, males showed higher discriminability index (M = 0.68; SD = 0.02) than females (M = 0.60; SD = 0.01); Difficulty (*F* (1,76) = 14.6; *p* < 0.001; *η*_*p*_^*2*^ = 0.16), the discriminability was worse in three boxes trials (M = 0.61: SD = 0.01) than in one box (M = 0.68; SD = 0.02); and Direction (*F*(1, 76) = 7.9; *p* = 0.006; *η*_*p*_^*2*^ = 0.11), clockwise trials showed higher discriminability index (X = 0.67: SD = 0.01) than anticlockwise trials (M = 0.61; SD = 0.02). No other main effects were found (*p* > 0.05).

The Difficulty x Age interaction reached significance (*F*(1, 76) = 19.7; *p* < 0.001; *η*_*p*_^*2*^ = 0.19). No other interaction effects were found (*p* > 0.05). There were significant differences in discriminability due to Difficulty only for old adults (*F*(1, 39) = 21; *p* < 0.001; *η*_*p*_^*2*^ = 0.32). The discriminability index was higher in the lower difficulty level (one box: M = 0.49; SD = 0.02) than in the higher difficulty condition (three boxes: M = 0.33; SD = 0.03) for old adults. On the other hand, there were no differences in discriminability in young adults (*p* > 0.05) with similar scores in the lower difficulty level (one box: M = 0.88, SD = 0.02) vs. the higher difficulty level (three boxes: M = 0.86; SD = 0.02).

The Direction x Difficulty interaction was also statistically significant (*F*(1, 76) = 6.8; *p* = 0.016; *η*_*p*_^*2*^ = 0.10). Analyses of the interaction showed that Direction affected performance only in the higher difficulty level (three boxes: *F*(1, 76) = 26; *p* < 0.001; *η*_*p*_^*2*^ = 0.21). The discriminability index was higher in clockwise trials (M = 0.68; SD = 0.03) than in anticlockwise trials (M = 0.54; SD = 0.02). Otherwise, the discriminability index was similar in lower difficulty level (one box: *p* > 0.05; clockwise: M = 0.69, SD = 0.01; anticlockwise: M = 0.68; SD = 0.01). No other interaction effects were found (p > 0.05).

## Discussion

Our study showed that spatial recognition was determined by age and sex of the participants, as well as task difficulty. In addition, the rotation direction between memory and recognition stimuli modulated the interaction between age, sex and task difficulty. Thus, young adults performed better than old adults, and differences were maximized in anticlockwise rotation between the memory and the recognition images. Effect sizes for relevant interactions were generally in the medium power range.

To conduct the study, we used the ASMRT, a spatial recognition task. This task demands memorizing the position of a certain number of stimuli (one or three green boxes) placed inside a virtual room. Afterward, ten recognition images are presented with a single green box, and participants are required to respond if the position of the green box matches the position of the box presented during the memorization image. Since the viewpoint was modified in the recognition phase regarding the original image memorized, correct decisions depend on an accurate representation of the room features, and understanding the flexible relationship between the stimuli available. Moreover, rotating viewpoints required scene manipulation in memory to align the novel perspective to the previous one^16^, which is a hippocampal-dependent function, as suggested in other studies^32,33^. Note that task demands in ASMRT are partially similar to the ones found in spatial perspective-taking tests, ascribed to the same recognition paradigm widely used in the field of mental rotation^69^. Thus, in the recognition phase of ASMRT, participants have to compare the image on the screen with a memorized image taken from other point of view, and the most accurate solution requires remembering and comparing selected positions of both images.

As suggested by our results, and in line with previous studies^53,54^, aging is affecting performance in this task. Young adults had a higher percentage of hits compared to old adults. Moreover, this latter group also presented more false alarms than young adults regardless of the condition (one or three boxes to memorize). The more errors performed by old adults when the number of boxes to be remembered increased clearly reflected how memorizing additional stimuli impacts performance. In addition, the rotation direction between memory and recognition images determined performance, especially in old adults. For this group, anticlockwise images were associated with fewer hits than clockwise images. However, in the case of young adults, they were unaffected by this effect. The tendency was different for false alarms or discriminability scores, where a clockwise superiority was found regardless of age group in the three boxes difficulty level.

The existence of a differential pattern between young and old adults is not new, as it is well known that spatial orientation is affected in normal aging^35,70,71^. Using the ASMRT task, a performance decline was described in old adults^63^. Specifically, 70–79 years old participants had higher errors compared to other 50–59 and 60–69 old adult groups when remembering and recognizing positions from different perspectives. It is worthy to stress that spatial recognition does not require navigating through the environment like in other spatial memory tasks^58^, but demands the management of an allocentric reference frame to solve the task. Participants have to understand the flexible relationship between the cues available in the virtual environment to determine positions. Other studies compared spatial perspective-taking abilities in young and old adults, demonstrating that age is related with decay of these skills^17,72^. When testing recognition of positions, it was demonstrated that young adults also outperformed old adults^73–75^. Thus, our current study aligns with these findings.

Like in other perspective-taking tasks^19,21^, the rotation direction determines performance. However, in our study, Hit scores were significantly modulated by the rotation direction only in old adults, which is a novel finding. Also, the rotation direction modulated false alarm scores in the most difficult condition (three boxes), again only in the old adults. Thus, performance was more altered when the recognition image was rotated anticlockwise. As suggested in mental rotation tasks, there is an important linear correlation between the orientations of both images and the time and number of errors^13^. Spatial recognition is also affected by the perspective rotation, with advantage of clockwise rotation^20^. Our findings imply that the clockwise superiority found with simpler stimuli also applies to complex environments. Spatial recognition is also modulated by perspective rotation with the advantage of clockwise rotation^20^. This clockwise rotation bias was identified by prior studies^23,76,77^, but, to our knowledge, this is the first time it showed a different pattern by age group.

This differential pattern could be explained at a neural level. The hippocampus and other regions related to spatial skills present a reduced volume in healthy old adults^78^. The hippocampal circuit seems essential for acquiring a viewpoint-independent judgment, necessary for building allocentric representations of the context^33^. These allocentric spatial representations are required for accurately recognizing places from different points of view, as demanded on the ASMRT. As demonstrated previously^79^, Alzheimer’s disease and mild cognitive impairment patients—where the medial temporal lobe function is presumably compromised—showed altered perspective-taking abilities. In addition to this not only the context representation would be less precise in old adults, but also their rotational skills. The right hemisphere would be especially involved in rotational processes^21^. Certain studies propose that aging-related brain decline would be more prevalent in the right hemisphere, combined with a less lateralized cognitive performance^80^. This would result in lower rotational performance, as shown in previous studies^37,81^. It is noteworthy to mention that old adults present a reduced capacity to quickly adapt their behavior to changing conditions^82^, making overcoming pre-existent cognitive biases more difficult^83^. Since the rotation direction of recognition images changed randomly from trial to trial in our task, the considerable difficulties in anticlockwise trials exhibited by old adults seem to support the clockwise bias theory^23^. However, as previously described, other studies relied on simpler stimuli, so more research with complex environments is needed.

On the other hand, sex differences were reported in different spatial memory tasks, when navigating real^84,85^ and virtual environments^35,62,65,86^ but also in tasks demanding other spatial skills like mental rotation^44,87^ or perspective-taking abilities^45,82,88^. Our study partially reaffirmed this trend. Sex differences appeared in the percentage of false alarms, specifically in the most difficult condition, three-boxes-anticlockwise trials, in young adults, demonstrating that men and women differed in their spatial recognition skills, with men outperforming women under greater difficulty demands. This is consistent with prior findings with the ASMRT^62^.

Considering these results, a certain level of difficulty seems to be necessary to detect inter-group differences, including sex-dimorphic performance in some spatial orientation tasks^62,68^. Low task difficulties lead to the appearance of ceiling effects, whereas high task demands are associated with floor effects in the scores. As reported in other tasks, the choice of an adequate difficulty level helps to disclose sex-based differences in spatial orientation tasks. Thus, in the ASMRT task, men outperformed women if they had to memorize three spatial positions, but not when only one location had to be remembered^63^, which is consistent with our results, where females had a higher percentage of false alarms than males.

Note that place recognition—like in the case of the ASMRT task—is favoured by the use of allocentric strategies, involving the knowledge about the relationships between different cues available in the context. Previous works reported sex differences in the type of strategies used by men and women in spatial orientation tasks. Men prefer an object-based strategy (allocentric), whereas women are more likely to use an egocentric-based strategy^45^. These sex-dimorphic preferences involved the recruitment of diverse brain regions, differing by sex^45,89^.

Finally, the effect of angular rotation^16^, usually found in the mental rotation field with simpler stimuli, could not be factored in the analysis due to ASMRT design, as angle differences between memory and recognition images were mainly distributed in the first and the eighth octants. Although this allowed us to control the angle impact on performance by the balanced distribution described previously, a different set of stimuli is needed in order to further disclose how this variable affects performance in complex environments, and how it interacts with rotation direction or how it can modify the chosen strategy depending on the degrees of rotation^24–26^. Also, as prior studies reported^20^, clockwise superiority could also be present in event-related potentials (ERP) measures, and hemispheric lateralization could also be relevant, resulting in differential activations depending on the rotation type^21^. A recent study also outlines differential activations according to sex factor in spatial recognition for complex stimuli^90^. Thus, to properly disclose if clockwise superiority is consistent for complex stimuli, and how it interacts with sex and age, subsequent studies integrating electroencephalography (EEG) measures need to be performed to extend our findings further beyond. Moreover, future studies should also consider how these variables affect response times, which could not be registered in our study due to the ASMRT methodology, which registered responses manually and could not measure precise response times^62^.

As a potential limitation, we should state that, even though the sample sizes were high for sex and age factors separately (n = 40 each), they were smaller when considering both factors together, so the lack of significance in this interaction should be interpreted cautiously. Overly, our study is a novel effort in assessing the rotation direction effect for complex environments and stimuli, signalled a potential prevalence of a clockwise bias, and proved how this effect is particularly associated with aging decline.

## Data Availability

The datasets generated during and/or analyzed during the current study are available and can be received from the corresponding author under reasonable request.
